# Effects of 8-week sensory electrical stimulation combined with motor training on EEG-EMG coherence and motor function in individuals with stroke

**DOI:** 10.1038/s41598-018-27553-4

**Published:** 2018-06-15

**Authors:** Li-Ling Hope Pan, Wen-Wen Yang, Chung-Lan Kao, Mei-Wun Tsai, Shun-Hwa Wei, Felipe Fregni, Vincent Chiun-Fan Chen, Li-Wei Chou

**Affiliations:** 1 Department of Physical Therapy and Assistive Technology, National Yang-Ming University, Taipei, Taiwan; 2 Spaulding Neuromodulation Center, Department of Physical Medicine & Rehabilitation, Spaulding Rehabilitation Hospital and Massachusetts General Hospital, Harvard Medical School, Boston, MA USA; 3 Department of Physical Medicine & Rehabilitation, School of Medicine, National Yang-Ming University, Taipei, Taiwan; 4 Division chief of General Rehabilitation, Department of Physical Medicine & Rehabilitation, Taipei Veterans General Hospital, Taipei, Taiwan; 5 Engineering Science, Loyola University Chicago, Chicago, IL USA

## Abstract

The peripheral sensory system is critical to regulating motor plasticity and motor recovery. Peripheral electrical stimulation (ES) can generate constant and adequate sensory input to influence the excitability of the motor cortex. The aim of this proof of concept study was to assess whether ES prior to each hand function training session for eight weeks can better improve neuromuscular control and hand function in chronic stroke individuals and change electroencephalography-electromyography (EEG-EMG) coherence, as compared to the control (sham ES). We recruited twelve subjects and randomly assigned them into ES and control groups. Both groups received 20-minute hand function training twice a week, and the ES group received 40-minute ES on the median nerve of the affected side before each training session. The control group received sham ES. EEG, EMG and Fugl-Meyer Assessment (FMA) were collected at four different time points. The corticomuscular coherence (CMC) in the ES group at fourth weeks was significantly higher (p = 0.004) as compared to the control group. The notable increment of FMA at eight weeks and follow-up was found only in the ES group. The eight-week rehabilitation program that implemented peripheral ES sessions prior to function training has a potential to improve neuromuscular control and hand function in chronic stroke individuals.

## Introduction

Stroke is one of the leading contributing factors to the loss of functional abilities and independence in daily life in adults^1^. The most common and widely observed impairment following stroke is motor impairment, which can be regarded as a loss or limitation of function in muscle control or movement^2–5^. Most stroke survivors later regain the ability to walk independently, but only fewer than 50% of them will have fully recovered upper extremity functions^6,7^. From a review focusing on motor recovery after stroke, it has been indicated that the recovery of both arm and hand function among subacute and chronic stroke survivors is limited in current neural rehabilitation settings^4^; therefore, additional management with activating plasticity before or during performing motor training is necessary for better motor recovery.

The fundamental principle of stroke rehabilitation is inducing brain plasticity by sensory or proprioceptive input in order to facilitate motor functions^8,9^. It has been demonstrated that strong sensory input can induce plastic changes in the motor cortex via direct or indirect pathways^10–17^. In this case, electrical stimulation (ES) that provides steady and adequate somatosensory input can be an ideal method of stimulating the motor cortex.

Recent studies using functional magnetic resonance imaging (fMRI) or transcranial magnetic stimulation (TMS) suggest that ES on peripheral nerves can increase motor-evoked potential (MEP)^18–20^, increase the active voxel count in the corresponding motor cortex^13^, and increase blood-oxygen-level dependent (BOLD) signals in fMRI, suggesting peripheral ES induced higher excitability and activation level of cortical neurons^21^. Since the expansion of the motor cortical area or increase in the excitability of neural circuits is associated with learning new motor skills^22–26^, clinicians should take advantage and assist patients with stroke on motor tasks training during this period of time. Celnik and colleagues^27^ found that the hand function of chronic stroke subjects improved immediately after two-hour peripheral nerve stimulation combined with functional training, and the effect lasted for one day. Based on previous studies, the ES that increases corticomuscular excitability may turn out to be an ideal intervention added prior to traditional motor training to “activate” the neural circuit, so that patients may get the most out of the training. According to a recent study that applied single session peripheral ES on post-stroke individuals, the corticomuscular coherence (CMC), which is the synchronization level between EEG and EMG, increased significantly and was accompanied by improvement in the steadiness of force output^28^.

To our knowledge, however, there is no study investigating the long-term effect of ES combined with functional training on both motor performance and cortical excitability. We targeted the median nerve because its distribution covered the dorsal side of index, middle, and half of ring finger and the palmar side of the first three fingers and half of the ring finger. Besides, median nerve is in charge of the flexion of the first three fingers, which combined they accounts for most of the functional tasks of hand. Therefore, the purpose of this pilot study was to preliminarily evaluate the effect of eight-week ES-combined hand functional training among chronic stroke patients based on CMC and motor performance. We followed up for four weeks after the intervention ceased and examined the lasting effect. We hypothesized that those who received intervention with ES would have better hand function and higher CMC than those who received intervention with sham ES. We also hypothesized that the effect would last for at least four weeks during our follow-up.

## Methods

This current study was a single-blind (subject) randomized placebo control trial. Blocked randomization was used to ensure equal number of subjects in each group.

### Subjects

Twelve chronic stroke survivors (1 female and 11 male subjects, age: 56.5 ± 9.5) were recruited for this study. All the participants were screened based on following criteria. The inclusion criteria were (1) first-ever cerebral cortical region involved chronic stroke, onset over a month, (2) able to perform active thumb flexion on the affected side with the scores of manual muscle test at least two points, and (3) at stable medical condition for intervention confirmed by a specialized physician. The exclusion criteria were (1) history of other neurological disorders, (2) cognitive impairment (Mini-Mental State Examination score < 24, MMSE)^29^, (3) unable to follow instructions, (4) contraindications of ES, and (5) under 20 years old. The protocol was approved by the Institutional Review Board at Taipei Veterans General Hospital, and the subjects gave their informed consent prior to participation in the experiments. All experimental procedures were performed in accordance with the Declaration of Helsinki (Clinical Registration number: NCT03277534; Date of registration: September 6, 2017).

### Experimental procedures

Eligible participants were randomly assigned into the ES-intervention group and the placebo control group. Both groups received treatment twice a week for eight weeks. The ES-intervention group received 40 minutes of ES^19,28,30,31^, while the placebo control group received 40-minute sham ES. The evaluation time points were set at the baseline, four weeks after, at the end of the eight-week intervention and four weeks after the intervention period ended. The evaluation included real-time electroencephalography (EEG) and electroencephalography (EMG) collection for CMC analysis and a functional test.

### ES parameters and functional training

The ES was delivered though 5 cm × 5 cm surface electrodes (S88 STIMULATOR, Grass technologies, RI, USA.) One of the electrodes was placed at volar side of elbow joint and the other placed 1 cm apart distally, just over the surface of median nerve. The subjects in ES group received 1 millisecond-rectangular pulse at 100 Hz with a 20-second on 20-second off cycle. The total ES intervention time is 40 minutes and the intensity was set at the highest tolerable level without pain or muscle twitch. These ES parameters have been shown to increase the cortical activities previously^19,28,30,31^. For the placebo control group, the surface electrodes were also placed on the aforementioned location, but no current was delivered. Subjects in the placebo control group were informed that due to the parameter settings of ES, it might not induce sensation. After 40-minute real or placebo ES, a certified physical therapist started 20-minute routine hand functional training for all subjects. They were asked to do tasks at different levels depending on their abilities, including picking up objects of different sizes and other instrumental activities of daily living tasks such as, using spoon, using scissors, writing, and so on. We documented the ES intensity for each subject. For each stimulation session, we started with the intensity that was last used and adjusted it according to the sensation of subject at the moment to ensure it was the highest tolerable sensory stimulation without pain and muscle twitch.

### EEG and EMG collection

The thenar eminence of the affected side were tested. The EEG and EMG signals were collected simultaneously while the subjects performed thumb isometric flexion at 50% maximal voluntary contraction (MVC) for 20 seconds. The subject’s forearm was supported on the table in the full supinated position. The thumb was secured in a ring-shape device, which was attached to a force transducer (FT10, Grass Technologies, RI, USA). EMG signals were recorded using surface electrodes (11-mm Ag-AgCl, 3.0 cm inter electrode distance) and were placed on the affected thenar eminence, proximal to first metacarpophalangeal joint and the ground electrode on styloid process. The force and EMG signals were amplified (P511 AC amplifier, Grass Technologies, RI, USA), and sampled (sampling rate 1000 Hz, CED Power1401 MK II, Cambridge Electronic Design Limited, UK) in real time and stored in a computer for later analysis. Verbal cues and visual feedback were given to help and encourage the subjects to maintain force output at 50% MVC. The subjects were asked to perform the trial twice with 1-minute break in between to avoid fatigue. We chose the trial with more stable force output for further comparison. The 16-channel EEG (actiCAP, Brain Products GmbH, Germany) was used to collect signals from bilateral sensorimotor and primary motor area including FC3, FC1, FCz, FC2, FC4, C3, C1, Cz, C2, C4, CP3, CP1, CPz, CP2, CP4, and Pz by 10–20 system where Cz is defined as the cross of midpoint between nasion and inion and between two ears. The EEG signals were amplified, filtered (1–100 Hz, 60 Hz notch) and sampled (2000 Hz) with BrainVision V-Amp (Brain Products GmbH, Germany.)

### Functional test

The Fugl-Meyer Assessment for Upper Extremity (FMA-UE)^32,33^ subset A to D were used for the functional test. There are 33 items in the assessment and the total score is 66 points. A higher score stands for better motor function of upper limb. Previous studies showed that FMA-UE provided high inter-rater and test-retest reliability, and is highly correlated to the Hemispheric Stroke Scale, which is a measurement of impairment^34–36^.

### Data management (CMC calculation)

Both EEG and EMG signals were further analyzed offline with MATLAB (The MathWorks, USA) to calculate the CMC based on the following equation^37^:

1 $$|{{\rm{C}}}_{xy}(f)|=\frac{{|\overline{{P}_{xy}(f)}|}^{2}}{\overline{{P}_{xx}(f)}\cdot \overline{{P}_{yy}(f)}}$$

C_xy_(f) is the coherence between x and y in frequency domain and can be calculated via Equation (1). In this study, P_xx_(f) and P_yy_(f) represent the power spectral density (PSD) of EEG or EMG in frequency domain; P_xy_(f) represents the cross PSD between EEG and EMG in frequency domain. The signals were analyzed within a 2048-sample epoch with 50% overlap. The length of the signal is 20 seconds and the resolution of frequency is 0.98 Hz.

Since only CMC values that exceed a theoretical threshold represent physiologically meaningful functional connections as described in an earlier study by Rosenberg^37^, we first calculated the theoretical threshold for each subject using the following Equation (2). In the equation, ${\rm{\alpha }}$ represents the confident interval, commonly being set at 95%, and *n* represents the number of epochs. Therefore, the length of the data can affect the theoretical threshold directly.

2 $$1-{(1-\frac{\alpha }{100})}^{\frac{1}{(n-1)}}$$

However, Rosenberg’s original equation did not take overlapping of the epoch into consideration during coherence analysis. Therefore, the equation was modified based on the overlapping of the epoch by a later study^38^.

3 $${r}_{th}^{2}(\alpha )=1-{\alpha }^{\frac{1}{\tilde{k}-1}}$$

In Equation (3), $\tilde{{\rm{k}}}$ represents the number of overlapped epochs and was calculated with the following Equation (4), where k represents the number of epochs without overlapping.

4 $$\tilde{k}=\frac{k}{{C}_{w}(D)}$$

Equation (4) was derived from a cumulative distribution function. To calculate ${C}_{w}(D)$, the following two equations (Equations 5 and 6) were used:

5 $${{\rm{C}}}_{w}(D)=1+2(\frac{k-1}{k}){\rho }_{w}^{2}(D)$$

6 $${\rho }_{w}(D)=\frac{{\sum }_{t=0}^{L-D-1}{w}_{L}(t){w}_{L}(t+D)}{{\sum }_{t=0}^{L-1}{w}_{L}^{2}(t)}$$

D represents the length that two signals were not overlapped and L represents the length of the epoch. In this study, we used the modified theoretical threshold to identify the physiological meaningful functional connection.

The CMC was evaluated between 0–70 Hz. However, since the motor task performed in this study was static isometric muscle contractions at 50% MVC, we focused on beta band CMC (13–30 Hz), as it is found to be more dominant during weak to moderate^39^, static^40^, and isometric^41^ muscle contractions. The summation of beta band CMC that exceeded the critical threshold of the electrodes in the affected hemisphere was calculated and referred to as the CMC value.

### Statistical analysis

All statistical analyses were performed using SPSS Statistics 22.0 (IBM, USA). Normal distribution of the data was checked by Shapiro-Wilk test. The primary outcomes of the study were CMC and FMA-UE. Intra-group comparisons of CMC and FMA-UE scores among four time points were conducted using the Friedman test. The Wilcoxon signed-rank test was used for post hoc analysis between two different time points. Inter-group comparisons of CMC and FMA-UE scores between the two groups at each time point were conducted using Mann–Whitney U test. All data are presented as mean values ± standard error of mean (±SEM) in figures or standard deviation (±SD) in text and the significant level was set at p < 0.05. For the post-hoc analysis between timing points, the significant level with Bonferroni adjustment was set at 0.017. The intra- and inter- power and effect size *r*^42^ for all between paired comparisons of each primary dependent variable were computed as well.

### Data Availability

The datasets generated and/or analyzed during the current study are available from the corresponding author on reasonable request.

## Results

**Figure 1:**
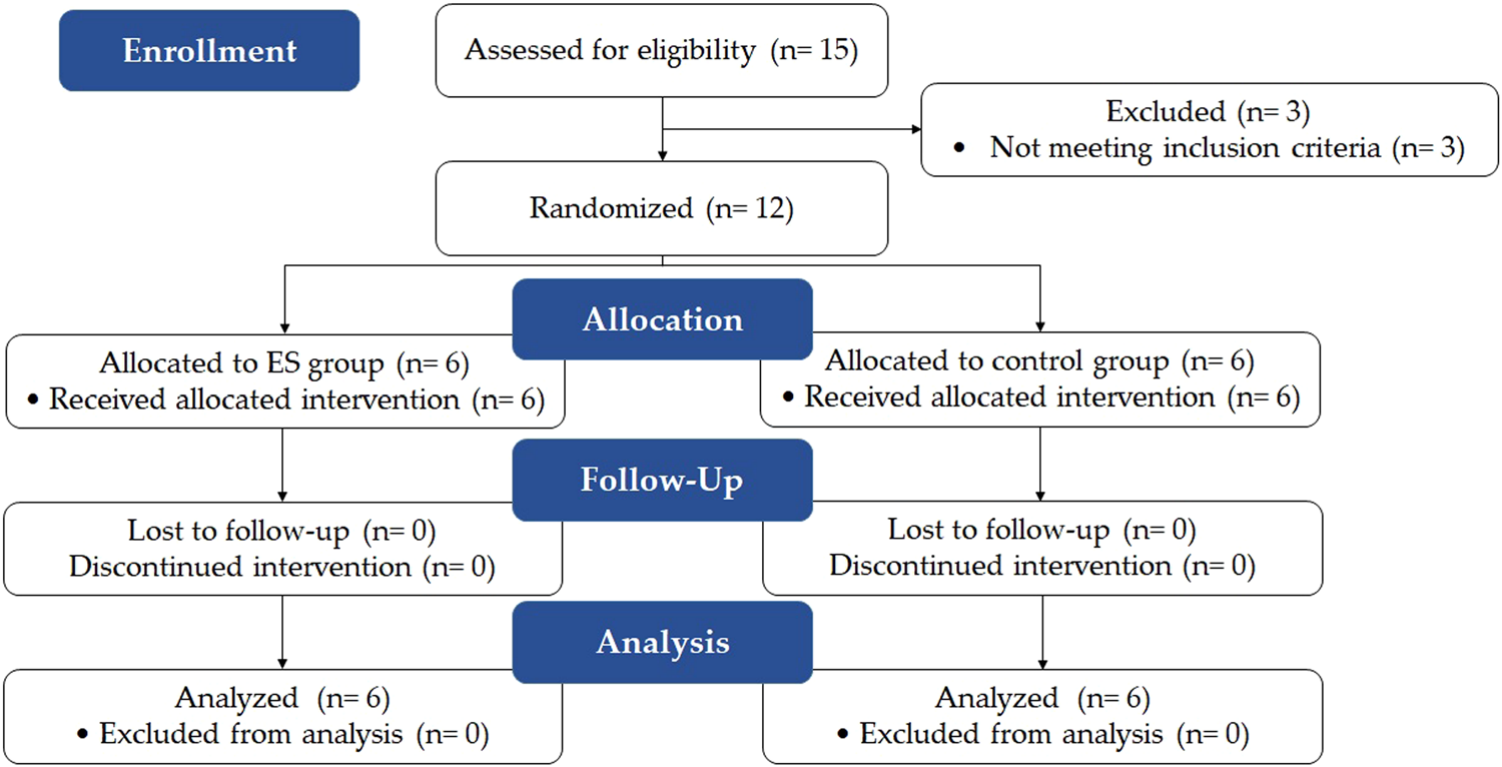
CONSORT flow chart. Fifteen stroke patients were screened for eligibility and three of them were excluded (for weak thumb flexion (manual muscle test = 0,0,1/5)). Twelve subjects were randomly allocated and all of them completed the intervention session and the follow-up assessment. The data collected from all subjects were analyzed.

All subjects (N = 12) received the intervention twice a week for eight weeks and finished all assessment sessions including follow-up. (Figure 1) The intervention was given based on the group assignment. The ES group received 40-min sensory level ES prior to motor training and the control group received sham-ES instead. The baseline of the two groups was not different in regards to demographic and clinical characteristics data (Table 1), CMC and FMA-UE (p > 0.05). Since the sample size was small and not normal distributed (p < 0.05), the non-parametric tests were performed for the following results.

**Table 1:** Demographic data

	ES	Control	P value
Age (yr.)	54.5 (6.9)	58.5 (10.1)	0.589
Height (cm)	168.5 (6.7)	169.3 (6.7)	0.589
Weight (kg)	62.8 (6.0)	65.5 (8.1)	0.394
Gender (M%)	100%	83.3%	0.500
Stroke type (H%)	66.7%	33.3%	0.284
Time since onset (mon.)	38.5 (25.8)	37.8 (15.0)	0.937
Affected side (R’t%)	66.7%	50%	0.284
Additional therapy (times/per week)	3.67 (1.8)	3.2 (1.5)	0.394
MMSE	29.0 (1.3)	27.2 (2.6)	0.485

Mean (SD); ES: electrical stimulation; yr.: years old; M: male; H: hemorrhage; R’t: right; MMSE: mini-mental state examination.

**Figure 2:**
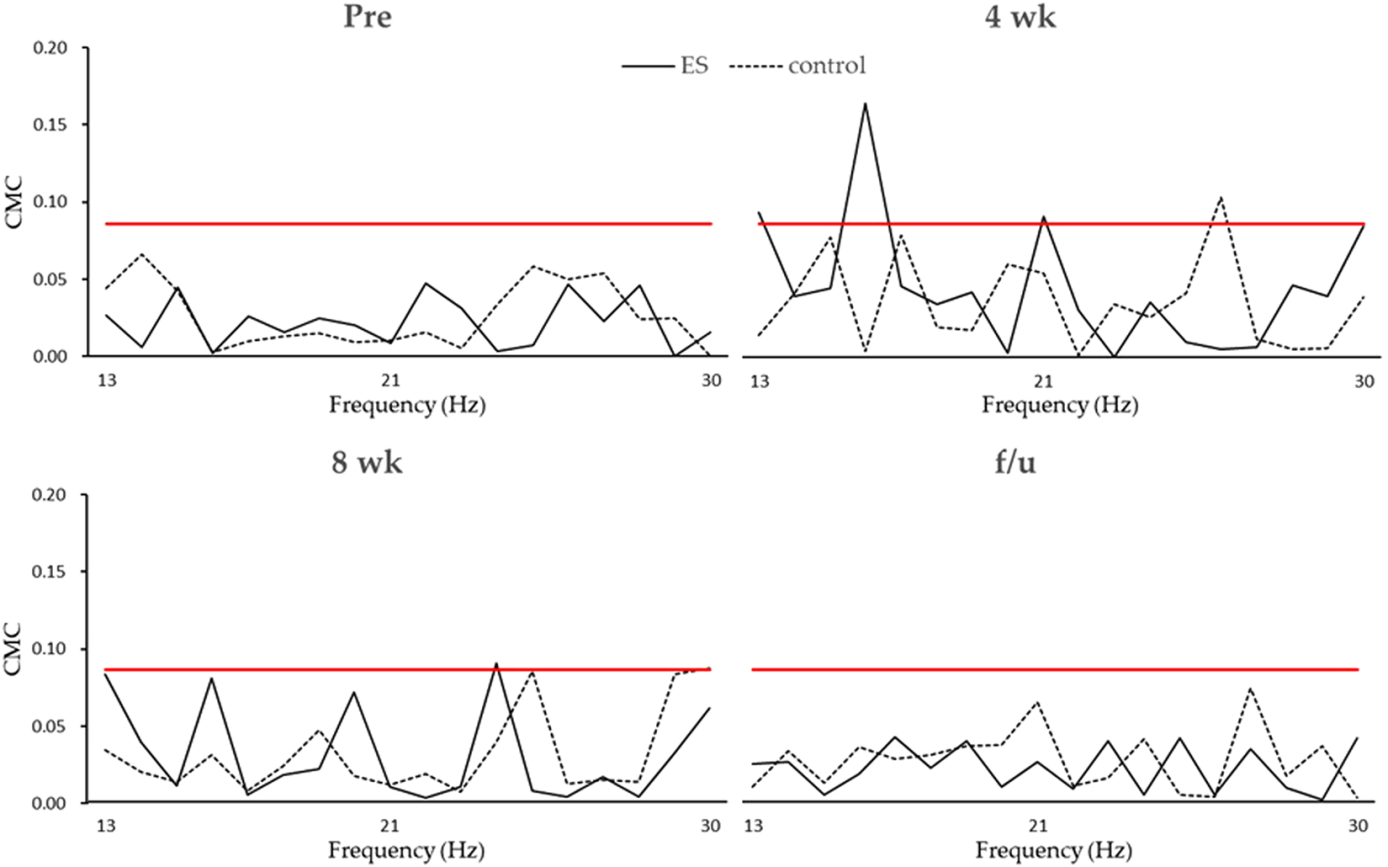
CMC of C2 electrode from typical subjects. CMC values before intervention (Pre), at the fourth week (4 wk), after completing 8 weeks of intervention (8 wk), and 4 weeks follow-up (f/u) for one subject in the ES group (dark line) and one subject in the control group (dashed line) were shown. The C2 electrode was chosen here because both subjects had right hemisphere stroke and C2 is the closet the right primary motor cortex hand area. The red line represents the critical threshold (0.0859) calculated by the equations described in Methods section. At the fourth week, the subject in the ES group had more area in beta band above the critical threshold, indicating the motor cortex had greater and physiologically meaningful functional connectivity with corresponding muscles.

**Figure 3:**
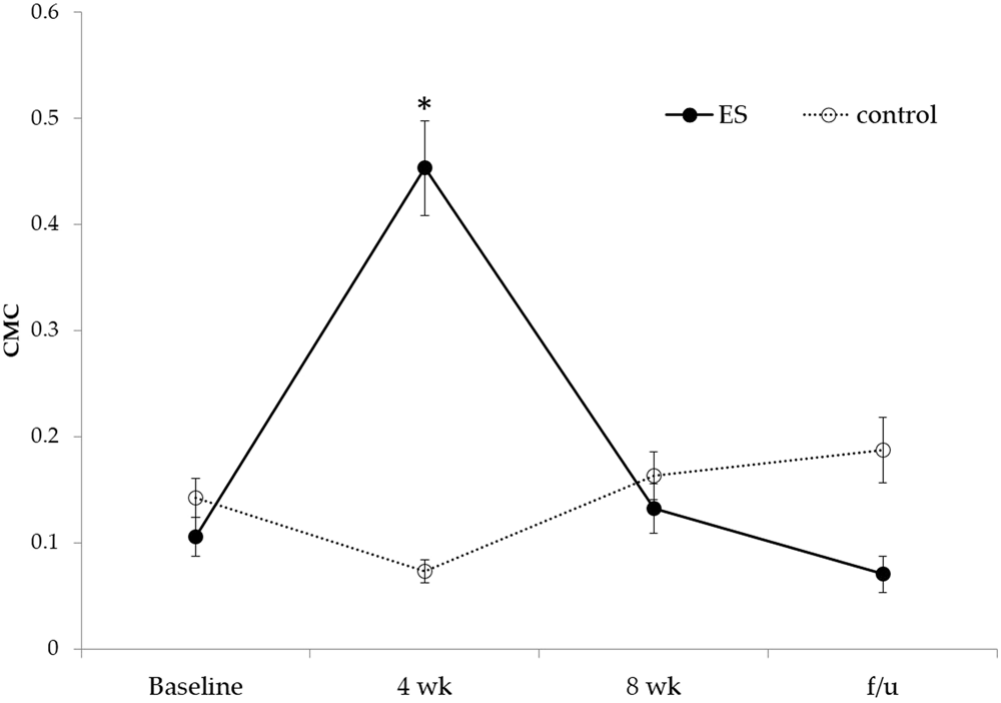
CMC values between the ES and control groups. The data were presented as mean and SE. The CMC value in ES group at 4-wk had a trend to be higher than baseline (p = 0.046), 8-wk (p = 0.028), and follow-up (p = 0.028) while no significance difference can be found in control group. Moreover, the CMC value at 4-wk in ES group was significantly higher (p = 0.004) than that in control group. CMC: corticomuscular coherence; ES: electrical stimulation; 4 wk: 4^th^ week; 8 wk: 8^th^ week; f/u: follow-up. *Significance between groups.

Figure 2 showed the C2 CMC plot of single subject from each group. The CMC values significantly differed among the four time points (Friedman test, p = 0.01). For the post-hoc analysis, the CMC in ES group at the 4^th^ week has a trend to be higher than baseline (p = 0.046, power = 0.940, effect size *r* = 0.785), 8^th^ week (p = 0.028, power = 0.939, effect size *r* = 0.899), and follow-up (p = 0.028, power = 0.966, effect size *r* = 0.899), while no difference was found in the control group. Moreover, the CMC at the 4^th^ week in the ES group was significantly higher (p = 0.004, power = 0.998, effect size *r* = 0.786) than that in control group (Fig. 3).

**Figure 4:**
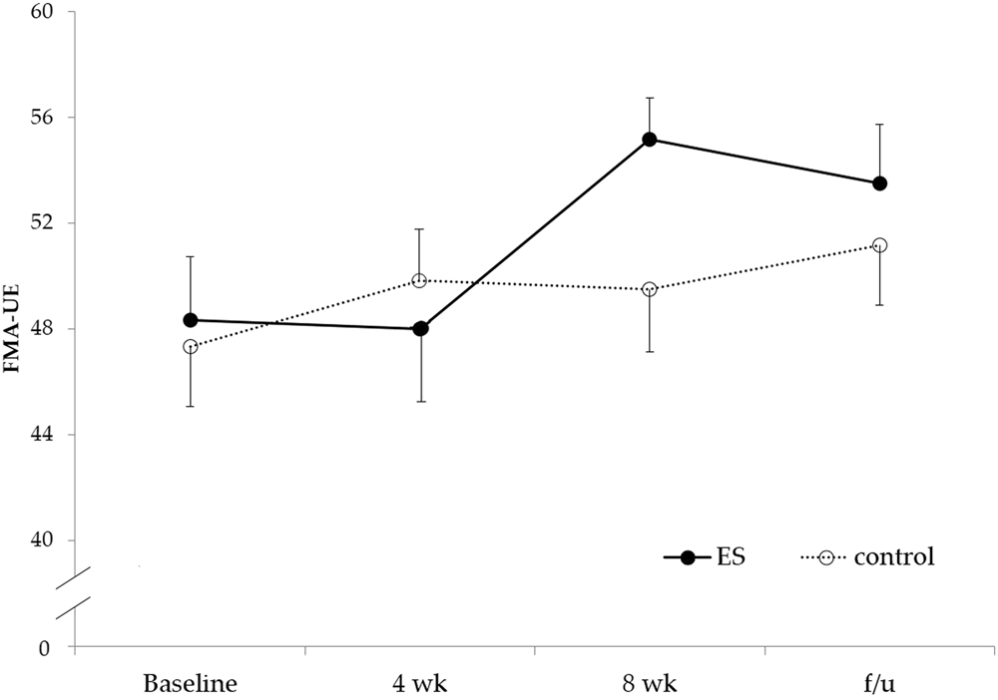
FMA-UE score between the ES and control groups. The data were presented as mean and SE. The FMA-UE scores in ES group at both 8-wk and follow-up had the trend to be higher compared to baseline (p = 0.042 and p = 0.042.) There was no significant change with time in control group. No statistically significance can be found between groups. FMA-UE: Fugl-Meyer Assessment for Upper Extremity; ES: electrical stimulation; 4 wk: 4^th^ week; 8 wk: 8^th^ week; f/u: follow-up.

The FMA-UE score significantly differed among the four time points (Friedman test, p = 0.035). For the post-hoc analysis, the notable increase of FMA-UE score at the 8^th^ week and during follow-up can only be found in the ES group as compared to the baseline (p = 0.042, power = 0.473, effect size *r* = 0.830 and p = 0.042, power = 0.374, effect size *r* = 0.830 respectively). No significant difference in FMA-UE between groups was found at any time point. On the other hand, the FMA-UE score of the control group only increased slightly without any significant change between each assessment session (Fig. 4).

We further conducted a Chi-square test with Fisher’s exact test to compare the number of subjects that improved more than 5 points or reached full score (66) between the two groups at each of the time points after treatment. This sub-analysis showed no significant difference in the proportion of people between the two groups at the 4^th^ week (p = 0.727), the 8^th^ week (p = 0.121), and the follow-up (p = 0.284). We further performed Pearson’s correlation analysis but no correlation between CMC increment at the fourth week and FMA-UE score at the eighth week (r = 0.234, p = 0.288).

## Discussion

In this proof of concept study, we confirmed our initial hypothesis that ES stimulation using an ON/OFF paradigm induces a significant effect in CMC and a significant improvement in motor function as indexed by FMA-UE scores. There are important novel insights from these data to be discussed: (1) duration and timing of behavioral effects; (2) changes in the neural marker (CMC).

Although not achieving statistical significance, it is important to note that 66.7% (4 out of 6) of the subjects in the ES group achieved a significant improvement of at least 5 points or achieved full score (66) in FMA-UE after 8-week intervention and the follow-up assessment, whereas only 1 subject in the control group achieved significant improvement. In fact, motor recovery after stroke is found to be limited, especially for the upper extremities^6,7^. Duncan and colleagues^43^ found that the subjects who had greatest motor recovery occurred at the first 30 days after stroke onset across different severity of initial impairments. However, those with moderate to severe initial impairments experienced additional motor recovery up to 90 days post-stroke. It was indicated that the motor recovery plateau occurred at around 12 weeks or three months after stroke^44,45^, yet there were still around 50% stroke survivors who suffered from upper extremity impairment at different levels^6^. This suggests that current rehabilitation programs are less effective for chronic stroke survivors. In this study, most of our subjects were at the chronic stage of stroke; however, those in the ES group still improved in motor function.

ES applied in the periphery elicits sensory inputs to modulate the excitability of motor cortex via two ascending pathways^46,47^. Our results indicate that additional sensory stimuli which facilitate cortical plasticity may have assisted motor re-learning even long after the onset of stroke. Although it has been suggested that peripheral ES can enhance the effectiveness of neurorehabilitation^48–50^, to our knowledge, this is the first study to monitor long-term effects of motor training combined with peripheral ES to induce cortical plasticity and motor recovery in chronic stroke patients. We found that in the ES group, the CMC in beta band increased significantly after four weeks of intervention and the FMA-UE score improved significantly after eight weeks of intervention. This improvement maintained throughout the four-week follow-up. We discuss below the timing of these changes, i.e., first neural marker changes and then behavioral changes.

In the ES group, the beta band CMC increased significantly after four weeks of intervention, but not for the eight-week assessment or during follow-up. Previous studies found that cortical plasticity is crucial for learning new motor tasks^22^, and greater cortical excitability was observed during motor re-learning^51,52^. Increased CMC in beta band suggested that during motor re-learning, the motor cortex has stronger connectivity between the central nervous system and muscles at the periphery. As the specific motor task being re-learned, less effort or attention will be needed to perform the specific motor task^52^. This phenomenon was observed as decreased or back-to-baseline CMC values after eight weeks of intervention in current study. In fact, the temporal sequence of results supports this CMC indication for motor learning. When we performed the EEG-EMG recording for CMC calculation, we recorded the actual force output level of thumb flexion at the same time. Therefore, we were able to observe the performance of sustained force output at 50% MVC. The accuracy (mean difference between target and actual force output) and steadiness (coefficient of variation between target and actual force output) of thumb flexion during EEG-EMG collection was calculated and the lower values stand for better performance. We found improvement of accuracy in ES group (6.9% ± 3.8% to 2.6% ± 1.9%) and improvement of steadiness as well (8.1% ± 5.5% to 6.2% ± 2.5%) even with lower CMC. On the other hand, those in control group even showed decreased performance with 2.3% ± 1.7% to 4.7% ± 3.6% for accuracy and 7.4% ± 2.4% to 12.8% ± 18.5% for steadiness. Hence, we can conclude that the motor task became less difficult for the subjects in ES group. The difference in trend change for CMC and FMA-UE score reflects the progress of motor re-learning. At the fourth week, as the subjects were still learning the new motor task, or establishing the best connections between the motor cortex and skeletal muscles, more attention was needed to perform the specific motor task. Therefore, the CMC value increased in order to maintain such motor performance. At the eighth week, the subjects acquired better motor function and neural planning (FMA-UE score) as they perform the same motor task during EEG-EMG collection, so less attention was required. As a result, less neural demand was needed between the motor cortex and muscles^53,54^. Even without significant correlation, we believe changes in CMC is linked to FMA-UE score as previously observed^28^. However, the correlation between CMC and motor function might not be strong in stroke patients^55^, and could be influenced by age, severity and area of stroke.

Our findings are in accordance with other studies that used sensorial enhancement as a method to engage the sensory system to enhance the effects of motor training strategies^49,56–64^. Other studies about motor recovery after stroke that also used FME-UA as outcome measurement reveal similar improvement in the score increment^56,60,61,65^. Quite a few other studies^66,67^ focused on the Constraint-Induced Movement Therapy (CIMT) in recent years. Yoon and colleagues^66^ compared the effect of CIMT, CIMT with mirror therapy, and control in subacute stroke subjects. The improvement in FMA-UE score was from 35.4 to 47.0, 47.9 to 53.3, and 32.7 to 37.0 for CIMT with mirror therapy, CIMT only, and control group, respectively. Based on a review, those who received CIMT improved 7.8 points (4.2 to 11.4) on average^67^.

Studies that applied additional electrical stimulation also found additional gains in motor recovery, though the targets for electrical stimulation were different. For example, for repetitive transcranial magnetic stimulation (rTMS), peripheral ES, and transcranial direct current stimulation (tDCS), positive results have been reported. Blesneag and colleagues^68^ applied low frequency rTMS over the primary motor cortex on acute ischemic stroke survivors and found that the FMA-UE scores of the subjects increased roughly from 29.6 at baseline to 42.8 after 45 days of intervention and reached 45 during a 90-day follow-up. Another study also applied rTMS over the primary motor cortex but focused on subacute to chronic stroke survivors. The authors found that the FMA-UE score increased from 28 to 30.9^69^. McDonnell and colleagues^56^ found that applying associative electrical stimulation of motor points on both hands prior to task-oriented training for three weeks showed improvement in FMA-UE score from 47.3 to 53.3. This particular study had intervention procedures similar to ours but with major differences in regard to ES parameters and the chronicity of the stroke (4.1 mon. vs. 38.5 mon.). Cha and colleagues^70^ applied tDCS on chronic stroke subjects (13.8 months post stroke) and found improvement of FMA-UE score from 20.5 to 48.7; this specific study revealed the greatest improvement among all, which may be due to the lower FMA-UE score at baseline. That is, the subjects had more room to improve after the intervention.

As aforementioned, a 5-point change in FMA-UE score indicates significant clinical change of motor function^71^. Therefore, the change of FMA-UE score in our study showed significant clinical improvement of upper extremity function and was similar to the results of other studies that provided motor training or electrical/magnetic stimulation as an approach for stroke intervention. The subjects of the ES group in this study were at more chronic phases (38.5 mon.) than other studies, but nevertheless, they still showed clinically significant improvement in the FMA-UE score. Hence, it can be assumed that chronic stroke survivors could benefit more from combined training. That is, by applying ES prior to functional training of upper extremities, we can facilitate the cortical plasticity of the corresponding region of the motor cortex and strengthen the connections between the region and the actuating muscles, so that patient’s corticomuscular pathway is “primed” for subsequent motor training^72^. The control group in this study showed trend of slight improvement in motor function. The results were in line with previous studies about the motor recovery in chronic stages; the most dramatic change took place within the first year after stroke and reached a plateau thereafter^73^.

The final point of discussion is the parameters of stimulation. There is intensive research on the optimal parameters of stimulation. In fact, it is a critical factor to determining the behavioral and neural effects of electrical stimulation^74–76^. Chen *et al*. showed that a variation of intensity can have a significant impact in induced neuroplasticity^77^; in fact, we used a protocol of ON/OFF to deliver ES that may also have been associated with significant improvements observed in this study. Another point to consider is potential home use. ES can be easily applied for home-based rehabilitation program. Therefore, the intervention in the current study could be appropriate rehabilitation at the chronic stage. The suitability at the acute phase requires future study to investigate the possible effects.

The main limitation of this pilot study was the small sample size. However, with only that limited sample size we showed clinically significant results that support our a priori hypothesis. Another limitation was that the CMC value was calculated based on one out of the two trials, however, the CMC values were stable between the trials within the same subject during the same assessment session. Thus, the findings here are important to encourage further research in this area. Future studies with more subjects and with different regimens of stimulation will be needed to confirm the results of this preliminary pilot study.

In conclusion, the results of our pilot study indicate that chronic stroke survivors who received eight weeks of ES prior to functional training can facilitate corticomuscular functional connectivity and hence improve upper limb function. Additionally, they bring important insights into the timing of neural and behavioral changes as well as support further studies applying peripheral electrical stimulation.
